# Estimation of Peanut Leaf Area Index from Unmanned Aerial Vehicle Multispectral Images

**DOI:** 10.3390/s20236732

**Published:** 2020-11-25

**Authors:** Haixia Qi, Bingyu Zhu, Zeyu Wu, Yu Liang, Jianwen Li, Leidi Wang, Tingting Chen, Yubin Lan, Lei Zhang

**Affiliations:** 1 College of Engineering, South China Agricultural University, Guangzhou 510642, China; qihaixia_scau@126.com (H.Q.); zby@stu.scau.edu.cn (B.Z.); 20193142028@stu.scau.edu.cn (Z.W.); liangyu@stu.scau.edu.cn (Y.L.); ljw@stu.scau.edu.cn (J.L.); 2 Guangdong Laboratory for Lingnan Modern Agriculture, Guangzhou 510642, China; ylan@scau.edu.cn; 3 College of Agriculture, South China Agricultural University, Guangzhou 510642, China; wangld@mail.iap.ac.cn (L.W.); chentingting@scau.edu.cn (T.C.); 4 College of Electronics Engineering, South China Agricultural University, Guangzhou 510642, China

**Keywords:** leaf area index, multispectral, remote sensing, density, vegetation index

## Abstract

Leaf area index (LAI) is used to predict crop yield, and unmanned aerial vehicles (UAVs) provide new ways to monitor LAI. In this study, we used a fixed-wing UAV with multispectral cameras for remote sensing monitoring. We conducted field experiments with two peanut varieties at different planting densities to estimate LAI from multispectral images and establish a high-precision LAI prediction model. We used eight vegetation indices (VIs) and developed simple regression and artificial neural network (BPN) models for LAI and spectral VIs. The empirical model was calibrated to estimate peanut LAI, and the best model was selected from the coefficient of determination and root mean square error. The red (660 nm) and near-infrared (790 nm) bands effectively predicted peanut LAI, and LAI increased with planting density. The predictive accuracy of the multiple regression model was higher than that of the single linear regression models, and the correlations between Modified Red-Edge Simple Ratio Index (MSR), Ratio Vegetation Index (RVI), Normalized Difference Vegetation Index (NDVI), and LAI were higher than the other indices. The combined VI BPN model was more accurate than the single VI BPN model, and the BPN model accuracy was higher. Planting density affects peanut LAI, and reflectance-based vegetation indices can help predict LAI.

## 1. Introduction

Leaf area index (LAI) is defined as half of the total green leaf area per unit of the horizontal ground surface area of the vegetation canopy [1]. LAI is used to estimate leaf cover and monitor and predict crop growth and yield [2,3], and it is a key parameter of photosynthesis, respiration, and transpiration in plants [4]. LAI is an important variable in many land surface models, and assimilating LAI-derived remote sensing data into crop models has improved estimates of biomass and yield [5]. The efficient and nondestructive monitoring of crop growth is essential for precise crop management and is key to modern precision agriculture [6]. Real-time LAI monitoring can provide information on crop health and nutrient status and assist fertilization and irrigation management efforts. LAI has traditionally been measured by in situ (destructive or optical) methods, which require significant time and human resources. Moreover, the sample-based measurements are spatially discontinuous [7,8]. Remote sensing techniques are often associated with green-related biophysical parameters, such as the crop chlorophyll content, vegetation biomass, or leaf area index, which directly or indirectly reflect crop vigor and photosynthetic capacity [9]. Since the development of remote sensing technology in the 1950s, satellites, manned aircraft, and ground-based spectral equipment have been used to monitor crop growth. However, these platforms have limitations. Satellite data are limited by altitude and orbit (i.e., clouds and suspended particles) and do not provide the spatial, temporal, or spectral resolution required for growth monitoring [10]. In recent years, with improvements in relevant technologies, satellite data have become available to meet monitoring needs. Manned aircraft are expensive to operate and require operators with relevant flight training. Ground-based spectral equipment is bulky and inefficient, inevitably causing damage to the crop canopies. In contrast, unmanned aerial vehicles (UAVs) offer an economical and efficient way to address the growing requirements for spatial, temporal, and spectral resolution [11]. UAVs have been used to acquire high-precision crop data, and crop monitoring via UAVs is common, including crop plot detection [12], fruit detection [13], crop yield [14], crop variable measurement [15], crop terrain mapping [16], and crop physiological parameter estimation [17]. Low-altitude remote sensing technology provides high temporal and spatial resolution, enabling the nondestructive, accurate, and timely estimation of leaf area index, crop growth, plant biomass, final crop yield, and other biophysiological parameters [18].

There are two general methods for estimating LAI from remotely sensed data: a process-based method and an empirical method based on the vegetation index (VI), also known as the VI method [19]. The process-based approach estimates LAI from a radiative transfer model, which was developed from remotely acquired canopy reflectance data [20]. Radiative transfer models simulate the (bidirectional) reflectivity of land surfaces through a series of physical or mathematical descriptions of the background (i.e., soil or snow surface), the object (i.e., the canopy or other surfaces), the physical and radiative properties of the atmosphere, and the geometry of the sun and sensors [21]. In contrast, the VI method establishes a statistical relationship between the remotely sensed VI and observed LAI values (hereafter referred to as the LAI-VI relationship) [22]. VI is the reflectance from two or more spectral bands and can be used to estimate the physiological and biochemical characteristics of vegetation, such as the LAI, biomass, and canopy chlorophyll content [23]. Su et al. constructed the Ratio Vegetation Index (RVI), Normalized Difference Vegetation Index (NDVI), and Optimized Soil-Adjusted Vegetation Index (OSAVI) to accurately monitor yellow rust in wheat using multispectral images based on UAVs footage [24]. Carlos et al. used UAVs for aerial crop monitoring to combine seven vegetation indices of rice growth in a multivariate regression model to estimate rice biomass [25]. Wang et al. developed a rice yield estimation model based on satellite images using field measurements and canopy reflection band ratios (NIR/RED, NIR/GRN), and successfully predicted rice yield over a large area [26]. Zarco et al. combined R515/R570 and Transformational Chlorophyll Absorb Reflectance Index (TCARI)/OSAVI narrowband indices to estimate leaf carotenoids with a hyperspectral camera on a UAV [27]. Vegetation indices provide quick and easily obtainable information for a better understanding of the underlying mechanisms of crops, and many VIs are closely related to LAI. The most common VIs are the simple ratio vegetation index [28] and NDVI [29]. However, there is an exponential relationship between NDVI and LAI, and NDVI is saturated when the aboveground biomass is too high [30]. When the LAI was 2 to 6, the reflectance in the near-infrared band was significantly higher than in the red band. When the reflectance in the near-infrared band exceeds 40%, the contribution of reflectance to NDVI is small [31]. Considerable efforts have been made to minimize the effects of the soil, including the Soil-Adjusted Vegetation Index (SAVI) [32], the optimized SAVI (OSAVI) [33], the improved SAVI [25], and the Atmospheric-Resistant Vegetation Index (ARVI) [28]. Steps have also been taken to improve the sensitivity of the vegetation indices at high LAI and to reduce atmospheric disturbance, such as the Enhanced Vegetation Index (EVI) [34,35], the Improved Delta Vegetation Index (MTVI2) [25], and the Wide Dynamic Range Vegetation Index (WDRVI) [36]. In addition to different VIs, the LAI-VI relationships use several mathematical equations, including linear, exponential, logarithmic, and polynomial [37,38].

Peanuts are grown worldwide as oil and cash crops. Peanut production in China and the United States accounts for approximately 70% of the total global production, and the global production and trade of peanuts is very important [39]. Planting density has a significant impact on crop growth status and is important for guiding high yielding crops. Currently, most research is devoted to the use of spectroscopic techniques to monitor crop physiological parameters to understand crop growth, but few studies have explored the relationship between spectra, growth parameters, and plant density. The prediction model usually includes physical and statistical models [40], and the statistical models for LAI prediction include parametric and nonparametric regression models. Parametric models are simple, but the accuracy of the prediction is limited by the selected band. The nonparametric regression method makes full use of the spectral information and has high accuracy and robustness. This study used two different peanut varieties planted at eight different densities to assess peanut LAI from multispectral data collected during the field experiments. The band sensitive to green plant LAI was selected to establish the vegetation index, and simple regression (SR) and backpropagation (BP) neural network models were constructed to achieve fast, accurate, and nondestructive prediction of the peanut LAI. A BP neural network (BPN) is a multilayer feed-forward network trained by error backpropagation, consisting of three levels: input, implicit, and output [41]. The BPN method is based on sample training to establish a model of vegetation index and LAI to estimate LAI. Fortin et al. used multispectral near-infrared and red bands as the inputs to a BPN model to achieve high-precision maize LAI predictions [42]. Peng et al. compared fuzzy logic and artificial neural networks as inverse models for predicting drought-tolerant soybean varieties and found that the artificial neural networks achieved 80% prediction accuracy [43]. The most important advantage of BPN over other nonlinear methods is that the neural networks can be approximated globally, with a high degree of accuracy. BPN modeling does not require prior assumptions, as it is largely determined by the characteristics of the data. BP neural networks are widely used in research and have important capabilities, such as fly linear mapping, generalization, and fault tolerance. However, artificial neural networks also have unstable training results and are highly influenced by the samples. The objectives of this study were (i) to explore the relationship between density and spectral, (ii) to identify the bands most sensitive to peanut density and LAI and construct vegetation indices, and (iii) to evaluate different prediction models and evaluate their performance for predicting peanut LAI.

## 2. Material and Methods

### 2.1. Test Design

The experimental area is located at the teaching and research base of South China Agricultural University in Zengcheng, China (23°09 N, 113°22′ E; altitude: 11 m). The area is characterized by a subtropical monsoon climate, with 1945 annual sunshine hours, an annual average temperature of 20–22 °C, and 1623.6–1899.8 mm of annual precipitation.

**Figure 1:**
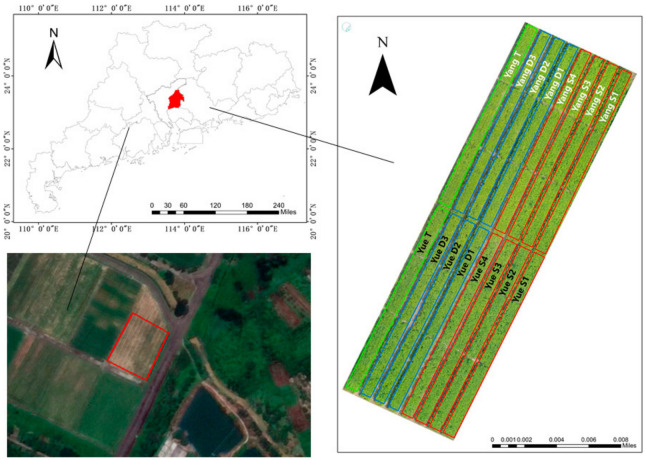
Location of the test area.

Peanut varieties Yueyou 45 and Yanghua No. 1 were selected as the test varieties and sown on 7 August 2019. The layout of the experimental plot is shown in Figure 1. The test field covered an area of 0.2 ha, containing 746 kg of compound fertilizer per ha and 896 kg of lime. The straddling width was 120 cm, the furrow width was 30 cm, there were 4 rows of peanuts in each row of fields, and the rows were spaced by 25 cm (10 cm spacing on the sides of the plot). There were 8 density treatments per peanut variety for a total of 16 plots: a single seed within an 8 cm row (S1), a single seed within a 10 cm row (S2), a single seed within a 12 cm row (S3), a single seed within a 20 cm row (S4), two seeds within a 16 cm row (D1), two seeds within a 24 cm row (D2), two seeds within a 20 cm row (D3), and three seeds within a 20 cm row (T). The plants were grown with the recommended fertilization and irrigation schemes.

### 2.2. Data Acquisition

#### 2.2.1. Multispectral Data Acquisition and Processing

A Parrot Sequoia 4-channel multispectral camera was mounted on a Parrot Bluegrass UAV to collect multispectral peanut data at key fertility stages, i.e., the seedling stage (7 September 2019), flowering stage (27 September 2019), pod-filling stage (26 October 2019), and maturity stage (17 November 2019). The setup included a 16 MP rolling shutter RGB camera (resolution: 4608 × 3456 pixels) and four 1.5 MP global shutter single-band cameras (resolution: 1280 × 960 pixels) in the following spectral bands: green (center wavelength = 550 nm, bandwidth = 40 nm), red (center wavelength = 660 nm, bandwidth = 40 nm), red-edge (center wavelength = 735 nm, bandwidth = 10 nm), and near-infrared (NIR, center wavelength = 790 nm, bandwidth = 40 nm) (Table 1) [6].

Radiation-calibrated images of a calibrated reflectance panel (MicaSense) were captured on the ground before each flight [43]. To obtain sufficient image resolution, the aircraft flew at 2.5 m/s at an altitude of 18 m above the ground, capturing 85% of the forward and lateral overlap images at 1.5 s intervals. During the flight, the Parrot Sequoia camera uses its built-in sunlight sensor and the calibrated reference board image to calibrate and correct the reflectivity of the captured images to minimize the amount of error [6]. After the flight, radiation correction of the multispectral images is performed using the following equation:

(1) $$R_{Target}=\frac{DN_{Target}}{DN_{C}}\cdot R_{C}$$

where $R_{Target}\;$is the reflectivity of the target, $DN_{Target}$ is the *DN* (Digital Number) value of the target, $DN_{C}$ is the *DN* value of the correction plate, and $R_{C}$ is the reflectivity value of the correction plate. The radiation calibration of the multispectral images from the UAV requires extraction of the corrective class DN values of the green, red, red-edge, and near-red band images separately. The radiation corrections are applied to each single-band image, which is then synthesized to obtain the multispectral image data.

Five multispectral image sets were generated after each UAV flight: green, red, red-edge, NIR, and Red, Green, and Blue (RGB). Image processing selected only the first four multispectral image sets because this study required VIs and four monochrome image reflections. Multispectral image stitching to form orthophoto images was performed in Pix4D mapper4.2 (Pix4D S.A., Lausanne, Switzerland) and included camera alignment, georeferencing, construction of dense point clouds, and orthogonal stitching [44].

#### 2.2.2. Collection and Processing of the Leaf Area Index

The leaf area index (LAI) was measured with a LAI-2200C plant canopy analyzer (Li-Cor Biosciences, Lincoln, NE, USA). This instrument is commonly used for in-field LAI measurements and is equipped with a fisheye optical sensor to measure the radiation above and below the canopy. Peanut LAI was measured below the plant after spectral data acquisition was completed. Five sample points were measured per plot, for a total of 80 LAI measurements, including GPS coordinates for each sample point, and the peanut LAI values were processed according to the time of measurement to remove anomalous data. The data were initially processed using Microsoft Excel to calculate the mean value of each plot sample point (*n* = 5), which was then used as the mean LAI for the plot.

### 2.3. Selection of the Vegetation Index

A vegetation index constructed from the combination of the red and near-infrared bands can enhance the effective information of the vegetation leaf area index [30]. In this study, 12 vegetation indices related to leaf area were selected for analysis (Table 2).

### 2.4. Prediction Model Construction and Verification Accuracy

This study used VIs as independent variables (LAI as dependent variable) for further modeling and comparative analysis. Two different modeling methods, SR and BPN, were used to predict peanut LAI, and the model accuracy was evaluated by the coefficient of determination (*R*^2^) and root mean square error (RMSE). High *R*^2^ and low RMSE values were indicative of high model accuracy and were calculated as:

(2) $$R^{2}=1-\frac{\sum_{j}^{M}{\left(y_{j}-\hat{y_{j}}\right)}^{2}}{\sum_{j}^{M}{\left(y_{j}-\bar{y_{j}}\right)}^{2}}$$

(3) $$\mathrm{RMSE}=\sqrt{\frac{1}{M}\sum_{j=1}^{M}{\left(y_{j}-\hat{y_{j}}\right)}^{2}}$$

where, $y_{j}$ and $\hat{y_{j}}$ are the measured and predicted values, $\bar{y_{j}}$ is the average of the measured values, respectively, and M is the number of samples.

The single and combined vegetation indices were used as input variables, and the measured LAI was used as an output variable to construct a single vegetation index neural network training model (S-BPN) and a combined vegetation index neural network training model (C-BPN), respectively. Using the MATLAB neural network toolbox, the number of iterative training sessions was set to 1000, the learning rate was set to 0.01, and the training function was used to iteratively train within a reasonable range of implicit layer nodes to identify the best network training effect. The number of implicit layer neurons was determined using [45] as follows:

(4) $$N_{h}=\sqrt{\left(m+2\right)N}+2\sqrt{\frac{N}{m+2}}$$

where $N_{h}\;$is the number of implicit layer neurons, *m* is the number of layers, and N is the number of input neurons.

## 3. Results

### 3.1. Spectral Data Processing

**Figure 2:**
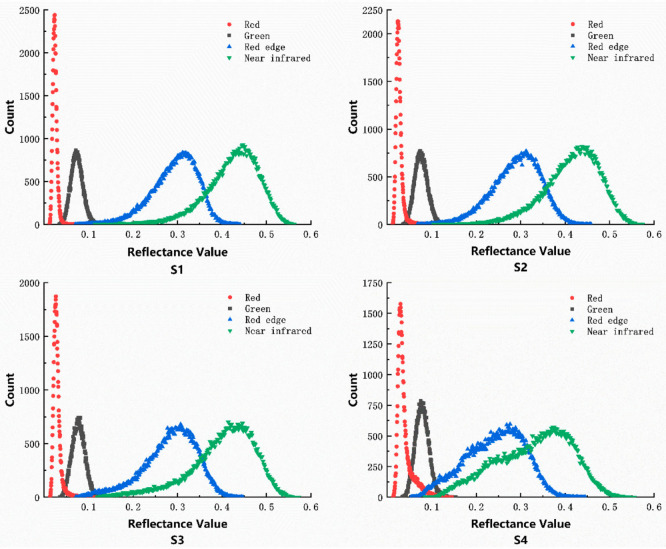
Histogram of the reflectance at different wavelengths for the peanut plants grown at different densities (S1, S2, S3, S4). *p* = 0.01, the correlation is significant.

The reflectance curves of the peanut plants grown at different densities were obtained using the region of interest marker tool in the ENVI software to select the markers for different density plots in the original multispectral images, using the single-seeded Yueyou 45 plants as an example (Figure 2). Plant density had a strong influence on the red light and near-red bands, and the peanut LAI increased with increasing plant density. This showed a relationship between the LAI and the red light and near-infrared bands. Therefore, these bands can be used to construct the vegetation index, which is consistent with previous studies.

### 3.2. Correlation Analysis between the Vegetation Indices and Measured LAIs

The 12 vegetation indices were correlated with the measured peanut LAI values at the four growth stages (Table 3). The vegetation indices were more strongly correlated with peanut LAI throughout the reproductive period than during any single growth stage.

**Figure 3:**
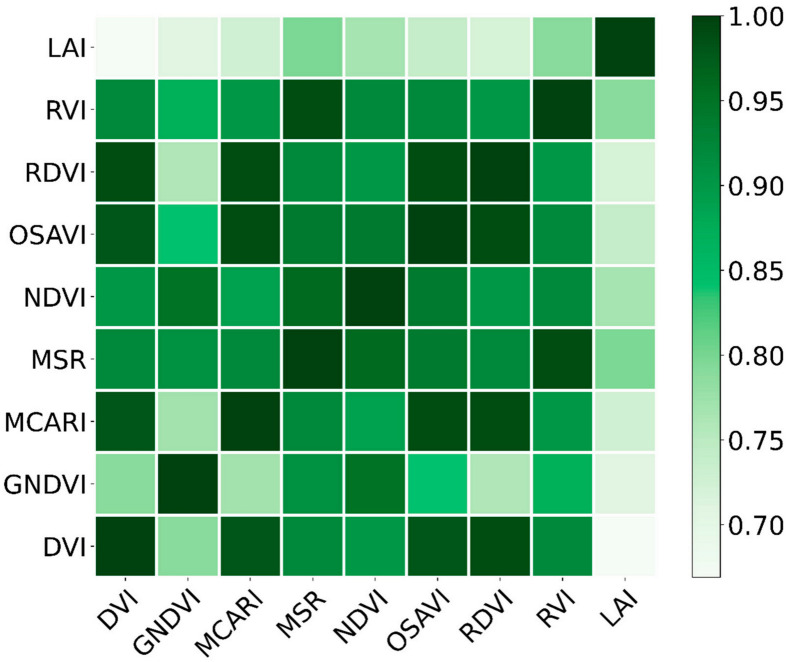
Correlations between the vegetation indices and peanut LAI during the whole growth period. *p* = 0.01, the correlation is significant.

Based on the above correlation analysis, eight VIs were selected: DVI, GNDVI, MCARI, MSR, NDVI, OSAVI, RDVI, and RVI. A correlation heat map of the VIs and the whole growth period was produced (Figure 3) and shows that MSR, NDVI, and RVI had the strongest correlations with LAI throughout the reproductive period (0.796, 0.767, and 0.789, respectively).

### 3.3. Peanut Leaf Area Index Analysis

**Figure 4:**
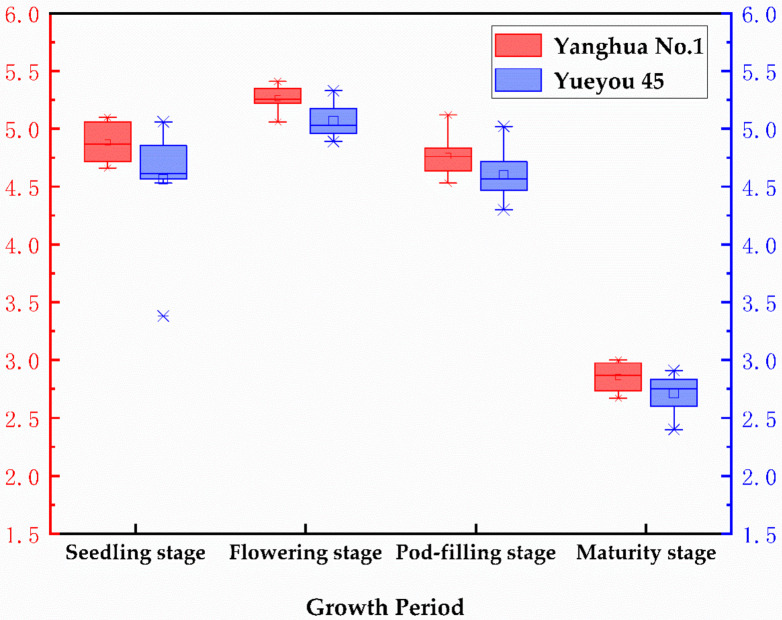
Changes in LAI throughout the growing period in two peanut varieties; *p* = 0.01, the correlation is significant.

**Figure 5:**
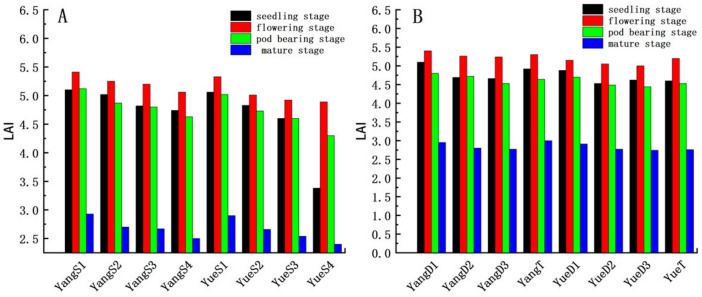
LAI for two peanut varieties under single sowing (**A**), and LAI for two peanut varieties under double sowing (**B**).

There were 320 measured values of LAI from the two peanut varieties across four growth stages. Five LAI data were collected from each plot, and the mean values were used as the mean LAI of the plot. The LAI of variety Yanghua 1 was higher than Yueyou 45 (Figure 4), and the LAI of both varieties peaked at the flowering stage. A comprehensive analysis of the different density plots for both varieties (Figure 5) showed that, throughout the growth stage, LAI was highest at the flowering stage, followed by the seedling stage, where the LAI was relatively low because of yellowing and fading leaves at the maturity stage. LAI was highest when the planting density was S1 and D1 (compared to the other densities). Figure 5A,B compares the single and double planting plots, respectively; the overall LAI of both varieties, regardless of the seeding method, decreased with decreasing density. Due to the early (seedling) stage, Yueyou 45 had a relatively low LAI because of poor growth in the S4 treatment.

### 3.4. Simple Regression Model

The SPSS software was used to randomly sample 64 peanut LAI measurements during the whole reproductive period; 50 were model samples, and 14 were test samples. The eight vegetation indices were fit with linear, logarithmic, power, and exponential models to select the most precise model for peanut LAI (Table 4).

Table 4 shows the predictive ability of the four regression models on peanut LAI; the first three single vegetation indices have similar accuracy to the inverse model, with *R*^2^ values of 0.773, 0.792, and 0.79, respectively. The fourth model is a multiple regression of the eight vegetation indices and measured LAI, which has the highest predictive accuracy (*R*^2^ = 0.83).

### 3.5. BP Neural Network Model

**Figure 6:**
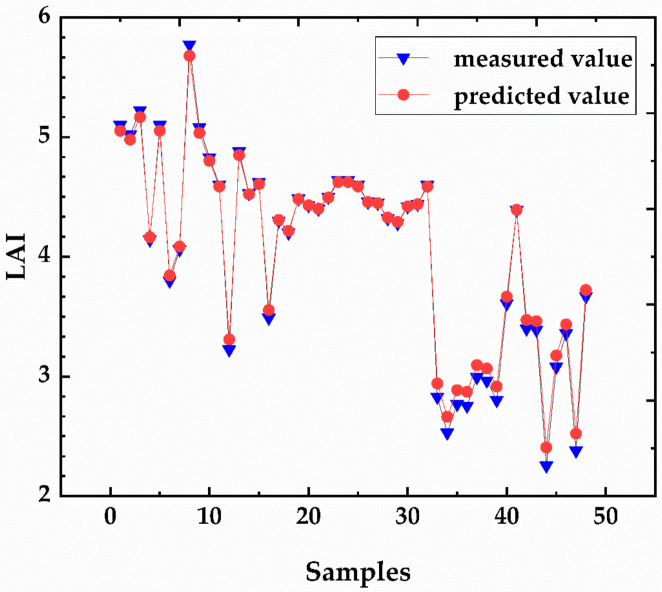
LAI-predicted results of the combined vegetation index neural network training (C-BPN) model.

In this study, the number of implicit layer neurons was set to 10, according to Equation (4). The training results are shown in Table 5, and the accuracy of the S-BPN models was mostly <0.9 (only the NDVI-BPN and RVI-BPN models had prediction accuracies >0.9). The C-BPN model predicts much better than the S-BPN models, reaching 0.968. Figure 6 compares the training results of the C-BPN model with the measured LAI values; the C-BPN predictions were very close to the observed values, suggesting good predictive ability.

### 3.6. Mapping the LAI Prediction

**Figure 7:**
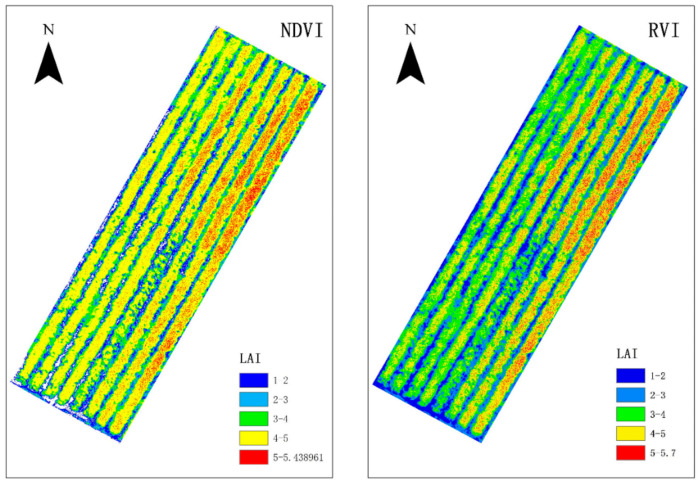
Prediction map of the RVI and NDVI vegetation indices for LAI.

The MSR, NDVI, and RVI vegetation indices were strongly correlated with peanut LAI. According to the simple regression models, NDVI-LAI and RVI-LAI (established in Section 3.4), the prediction accuracy of peanut LAI reached 0.773 and 0.79, respectively. The NDVI and RVI prediction filling plots of peanut flowering LAI using the ENVI 5.2 software are shown in Figure 7.

## 4. Discussion

Improving peanut yield has been the focus of research in the peanut field, and in the traditional cultivation model, the sowing density is too high for optimal peanut production and seedling yield [39]. Variety characteristics and planting densities are related to the physiological morphology of the peanut plant and affect crop yield. Thus, optimal planting densities can improve the group leaf area index, which can enhance peanut production per unit area. This study showed that peanut LAI tended to first increase and then decrease during the reproductive process, and LAI peaked at the flowering stage. Peanut LAI also tended to increase with density (either single or double sowing) and was highest in the S1 and D1 treatments compared to the other density treatments throughout the reproductive period. Appropriate sowing densities are conducive to the regulatory relationships between individual plants and groups, and optimal group structure contributes to high crop yield [39].

The leaf area index is a key variable linking remote sensing observations to the quantification of agroecosystem processes [46]. In agroecosystem studies, LAI is commonly used to estimate photosynthesis, evapotranspiration, crop yield, and many other physiological processes [19]. Since LAI is functionally linked to canopy spectral reflectance, it is widely used in remote sensing prediction studies [30]. Most VIs used for LAI estimation combine reflectance in the visible (RGB) and near-infrared (NIR) bands [30]; the visible wavelengths help to control background soil disturbance effects, and the NIR wavelengths allow a large dynamic detection range [47]. In this study, we selected 12 different vegetation indices that are correlated with LAI and established simple regression (SR) and artificial neural network models (BPN). The VIs had good predictive capacities for peanut LAI, among which MSR, RVI, and NDVI were the best. The multivariate regression model with eight VIs had a higher predictive accuracy for LAI (*R*^2^ = 0.83) than the regression models with single VIs. Furthermore, the combined vegetation index neural network training model (C-BPN) had a higher prediction accuracy (*R*^2^ = 0.968) than the single vegetation index neural network training model (S-BPN). Overall, the BPN models were more accurate than the SR models. In addition, more high-precision prediction models have yet to be further explored. However, to ensure that these spectral indices can be used as a general tool for green vegetation leaf area index prediction, further experimental validation is required for other plant species in different geographic and climatic regions. The methods presented here represent important advances in the nondestructive monitoring of peanut LAI that can aid accurate predictions of crop content during growth [46].

## 5. Conclusions

The results of this study show that plant density has a significant effect on peanut LAI. Generally, there was a positive correlation between planting density and LAI. Plant density also affected the red light (RED) and near-infrared (NIR) bands, suggesting that these are the sensitive bands of LAI. The simple regression (SR) prediction model and the BP neural network (BPN) prediction model of peanut LAI achieved good predictive results. The neural network model with the combined vegetation indices (C-BPN) had the best prediction effect and predicted peanut LAI with the highest accuracy. Thus, the C-BPN model can be used for real-time LAI monitoring during peanut production.

## Figures and Tables

**Table 1:** Specifications of the multispectral sensor used in this study.

Band Number	Band Name	Center Wavelength (nm)	Bandwidth FWHM (nm)	Definition
1	Green	550	40	1.4 MP
2	Red	660	40	1.4 MP
3	Red-Edge	735	10	1.4 MP
4	Near-Infrared	790	40	1.4 MP

**Table 2:** Definition of the selected vegetation indices (VIs).

Vegetation Index Equation	References
$\mathrm{Ratio}\;\mathrm{Vegetation}\;\mathrm{Index}\;\left(\mathrm{RVI}\right)=\mathrm{NIR}/\mathrm{RED}$	Jordan et al., 1969
$\mathrm{Normalised}\;\mathrm{Difference}\;\mathrm{Vegetation}\;\mathrm{Index}\;\left(\mathrm{NDVI}\right)=\left(\mathrm{NIR}-\mathrm{RED}\right)/\left(NIR+RED\right)$	Peñuelas et al., 1997
$\mathrm{Soil}-\mathrm{Adjusted}\;\mathrm{Vegetation}\;\mathrm{Index}\;\left(\mathrm{SAVI}\right)=1.5\left(\mathrm{NIR}-\mathrm{RED}\right)/\left(\mathrm{NIR}-\mathrm{RED}+0.5\right)$	Haboudane et al., 2004
Renormalized Difference Vegetation Index RDVI=NIR−RED/NIR+RED12	Roujean et al., 2005
$\begin{array}{c} \mathrm{Green}\;\mathrm{Normalized}\;\mathrm{Difference}\;\mathrm{Vegetation}\;\mathrm{Index}\;\left(\mathrm{GNDVI}\right)\\ =\left(\mathrm{NIR}-\mathrm{GREEN}\right)/\left(\mathrm{NIR}+\mathrm{GREEN}\right) \end{array}$	Gitelson et al., 1996
Modified Red **-** Edge Simple Ratio Index MSR=NIR/RED−1/NIR/RED+112	Chen et al., 1996
$\mathrm{Difference}\;\mathrm{Vegetation}\;\mathrm{Index}\left(\mathrm{DVI}\right)=\mathrm{NIR}-\mathrm{RED}$	Becker et al., 1988
Normalized Difference Red **-** $\mathrm{Edge}\;\mathrm{Index}\left(\mathrm{NDRE}\right)=\left(\mathrm{NIR}-\mathrm{RedEdge}\right)/\left(\mathrm{NIR}-\mathrm{RedEdge}\right)$	Gitelson et al., 1994
Red **-** $\mathrm{edge}\;\mathrm{chlorophyll}\;\mathrm{index}\;\left({\mathrm{CL}}_{\mathrm{RE}}\right)=\mathrm{NIR}/\mathrm{RedEdge}-1$	Gitelson et al., 2005
$\mathrm{Optimized}\;\mathrm{SAVI}\;\left(\mathrm{OSAVI}\right)=\left(1+0.16\right)\left(\mathrm{NIR}-\mathrm{RED}\right)/\left(\mathrm{NIR}+\mathrm{RED}+0.16\right)$	Rondeaux et al., 1996
$\begin{array}{c} \mathrm{Modified}\;\mathrm{Chlorophyll}\;\mathrm{Absorption}\;\mathrm{in}\;\mathrm{Reflectance}\;\mathrm{Index}\;\left(\mathrm{MCARI}\right)\\ =1.2*\left[2.5*\left(\mathrm{NIR}-\mathrm{RED}\right)-1.3*\left(\mathrm{NIR}-\mathrm{GREEN}\right)\right] \end{array}$	Daughtry et al., 2000
$\begin{array}{c} \mathrm{Modified}\;\mathrm{Triangular}\;\mathrm{Vegetation}\;\mathrm{Index}\;1\;\left(\mathrm{MTVI}1\right)\\ =1.2*\left[1.2*\left(\mathrm{NIR}-\mathrm{GREEN}\right)-2.5*\left(\mathrm{RED}-\mathrm{GREEN}\right)\right] \end{array}$	Haboudane et al., 2004

**Table 3:** Correlations between the vegetation indices and peanut leaf area index (LAI) at each growth stage.

Growth Stage	Vegetation Index											
	${\mathrm{CI}}_{\mathrm{RE}}$	DVI	GNDVI	MCARI	MSR	MTVI1	NDRE	NDVI	OSAVI	RDVI	SAVI	RVI
Seedling stage	0.124	0.467	0.383	0.422	0.481	0.422	0.232	0.506	0.529	0.515	0.513	0.426
Flowering stage	0.28	0.643	0.545	0.627	0.554	0.35	0.326	0.525	0.631	0.625	0.61	0.582
Pod-filling stage	0.548	0.389	0.524	0.377	0.644	0.377	0.506	0.918	0.481	0.443	0.421	0.641
Maturity stage	0.062	0.178	0.231	0.182	0.319	0.156	0.064	0.281	0.2	0.203	0.188	0.341
Whole growth period	0.394	0.669	0.709	0.725	0.796	0.659	0.438	0.767	0.739	0.72	0.714	0.789

Note: DVI, Difference Vegetation of Index; GNDVI, Green Normalized Difference Vegetation Index; MCARI, Modified Chlorophyll Absorption in Reflectance Index; MSR, Modified Red-Edge Simple Ratio Index; NDVI, Normalized Difference Vegetation Index; OSAVI, Optimized SAVI; RDVI, Renormalized Difference Vegetation Index); RVI, Ratio Vegetation Index. *p* = 0.01, the correlation is significant.

**Table 4:** The optimal inverse model and accuracy check of peanut LAI with each vegetation index construction.

Model	Equation	*R* ^2^	RMSE
NDVI-LAI	$y=5.919x^{1.872}$	0.773	0.407
MSR-LAI	y=0.9586x+1.197	0.792	0.389
RVI-LAI	$y=1.289x^{0.4629}$	0.790	0.390
8 VIs	LAI = 52.95DVI − 2.12GNDVI + 0.3MCARI + 7.31MSR + 11.12NDVI − 66.48RDVI − 0.77RVI	0.830	0.376

Note: R^2^, determination coefficient; RMSE, root mean square error. *p* = 0.01, the correlation is significant.

**Table 5:** Comparative analyses of the accuracies of the backpropagation (BP) neural network models for different input parameters.

Model	*R* ^2^	RMSE
DVI-BPN	0.857	0.127
GNDVI-BPN	0.875	0.2
MCARI-BPN	0.863	0.235
MSR-BPN	0.896	0.145
NDVI-BPN	0.923	0.124
OSAVI-BPN	0.873	0.165
RDVI-BPN	0.885	0.144
RVI-BPN	0.927	0.059
All VIs-BPN	0.968	0.165

Note: VIs-BPN is an inverse model of the established vegetation index versus the measured LAI. *R*^2^ indicates the prediction accuracy of the established neural network model on peanut LAI, and RMSE indicates the root mean square error of the model.
